# Evaluating the amoeba thioredoxin reductase selenoprotein as potential drug target for treatment of *Acanthamoeba* infections

**DOI:** 10.1016/j.ijpddr.2024.100564

**Published:** 2024-09-14

**Authors:** Alvie Loufouma-Mbouaka, Attila Andor, David Leitsch, Jorge Pérez-Serrano, Elias S.J. Arnér, Julia Walochnik, Tania Martín-Pérez

**Affiliations:** a Institute of Specific Prophylaxis and Tropical Medicine, Center for Pathophysiology, Infectiology and Immunology, Medical University of Vienna, 1090, Vienna, Austria; b Department of Selenoprotein Research and the National Tumor Biology Laboratory, National Institute of Oncology, Budapest, Hungary; c Division of Biochemistry, Department of Medical Biochemistry and Biophysics, Karolinska Institutet, Stockholm, Sweden; d Department of Biomedicine and Biotechnology, Faculty of Pharmacy, University of Alcalá, 28871, Alcalá de Henares, Spain

**Keywords:** *Acanthamoeba castellanii*, Redox system, Thioredoxin reductase, Selenoprotein, Auranofin, Thioredoxin reductase inhibitor

## Abstract

The genus *Acanthamoeba* comprises facultative pathogens, causing *Acanthamoeba* keratitis (AK) and granulomatous amoebic encephalitis (GAE). In both diseases, treatment options are limited, and drug development is challenging. This study aimed to investigate the role of the large thioredoxin reductase selenoprotein of *Acanthamoeba* (AcTrxR-L) as a potential drug target assessing the effects of the thioredoxin reductase inhibitors auranofin, TRi-1, and TRi-2 on AcTrxR-L activity and on the viability of *Acanthamoeba* trophozoites. Recombinant expression and purification of AcTrxR-L as a selenoprotein allowed assessments of its enzymatic activity, with reduction of various substrates, including different thioredoxin isoforms. Auranofin demonstrated potent inhibition towards AcTrxR-L, followed by TRi-1, and TRi-2 exhibiting lower effectiveness. The inhibitors showed variable activity against trophozoites in culture, with TRi-1 and TRi-2 resulting in strongly impaired trophozoite viability. Cytotoxicity tests with human corneal epithelial cells revealed lower susceptibility to all compounds compared to *Acanthamoeba* trophozoites, underscoring their potential as future amoebicidal agents. Altogether, this study highlights the druggability of AcTrxR-L and suggests it to be a promising drug target for the treatment of *Acanthamoeba* infections. Further research is warranted to elucidate the role of AcTrxR-L in *Acanthamoeba* pathogenesis and to develop effective therapeutic strategies targeting this redox enzyme.

## Highlights

• Recombinant AcTrxR-L was expressed, purfied and confirmed as a selenoprotein with enzymatic activity.
• Enzyme kinetic assays showed AcTrxR-L's ability to reduce various substrates, including different thioredoxin isoforms.
• Auranofin, TRi-1 and TRi-2 compounds inhibited AcTrxR-L, indicating their potential as future amoebicidal agents.
• Auranofin, TRi-1 and TRi-2 impaired trophozoite viability in culture.

## Graphical abstract

**Figure:**
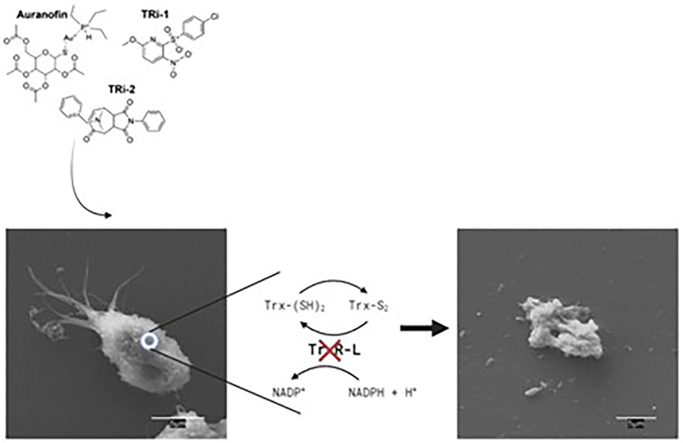

## 1 Introduction

*Acanthamoeba* species are cosmopolitan free-living amoebas found in water, soil and air environments (Siddiqui and Khan, 2012). The amoebas of this genus are opportunistic pathogens which can cause *Acanthamoeba* keratitis (AK), a sight-threatening corneal inflammation, which affects mostly healthy individuals wearing contact lenses, and granulomatous amoebic encephalitis (GAE), a fatal infection of the central nervous system, occurring mostly in immunocompromised individuals. Moreover, they can cause disseminated infections, affecting the skin, sinuses, lungs and other organs, also commonly observed in immunocompromised individuals (Marciano-Cabral and Cabral, 2003). AK is more common than the two other infections and represents 2% of corneal infections worldwide (List et al., 2021) and its prevalence is increasing (Lorenzo-Morales et al., 2015; Nielsen et al., 2020; Randag et al., 2019). This is due to the continual increase in the use of contact lenses, associated with inadequate contact lens care and hygiene (Maycock and Jayaswal, 2016). Treatment for AK lacks international standardization and national guidelines, as well as specific drugs against these amoebas. The current standard regime relies on broad-spectrum antiseptics, such as chlorhexidine (CLX) or polyhexamethylene biguanide (PHMB), often in combination with propamidine isethionate (Randag et al., 2019). Moreover, AK therapy can be challenging due to *Acanthamoeba*'s highly resistant cyst stage, which frequently necessitates prolonged, aggressive, and demanding treatment with severe side effects. In addition, recurrence of AK is possible when the treatment fails to eradicate the cysts (Dart et al., 2009). Despite advancements in the past years (Roberts and Henriquez, 2010), there is a need for the development of drugs that combat the cysts or inhibit encystment. Principally, as *Acanthamoeba* infections are very rare, only limited resources have been devoted to understanding the parasite's biology and to the development of new treatments (Angelici et al., 2021). Consequently, many details of *Acanthamoeba*'s cell biology and metabolism remain unknown. Data from more extensively studied protozoan parasites have revealed enzymes in redox networks as promising drug targets due to their crucial role in maintaining cellular redox homeostasis (Fata et al., 2022; Kuntz et al., 2007; Pal and Bandyopadhyay, 2012). Targeting these enzymes offers a feasible avenue for developing novel antiparasitic drugs. Examples include benznidazole and nifurtimox inhibiting trypanothione reductase (TryR) in trypanosomes (De Rycker et al., 2023; Kourbeli et al., 2021), responsible for diseases like sleeping sickness and Chagas disease, as well as artemisinin derivatives increasing ROS levels and inhibiting glutathione reductase (GR) in *Plasmodium falciparum*, the etiologic agent of malaria tropica (Egwu et al., 2021, 2022). Parasite selenoprotein thioredoxin reductases may be particularly suitable drug targets (Gencheva et al., 2022).

*Acanthamoeba* belongs to the Amoebozoa and thus is phylogenetically more closely related to humans than most other protozoan pathogens (Roberts and Henriquez, 2010). This raises concerns about potential side effects or toxicity of new drugs designed to treat *Acanthamoeba* infections in humans. Conversely, these similar molecular pathways might facilitate repurposing medications initially designed for human cancer treatment. Structure-drug design is a potentially useful approach to repurposing these drugs (Ondarza, 2007). It compares the three-dimensional structures of target enzyme active sites in the parasite and the host. Through this technique, compounds that specifically bind to the parasites’ enzymes can potentially be designed (Klebe, 2000).

For these reasons, a recent study by Leitsch et al. (2021) investigated the thioredoxin and glutathione system in *A. castellanii*. This study revealed the characteristics and the roles of the respective components in the response to oxidative stress. *A. castellanii* expresses two types of thioredoxin reductases (TrxRs): a small form of approximately 35 kDa (AcTrxR-S) also found in bacteria, archaea, plants and other unicellular eukaryotes, and a large selenoprotein form of approximately 55 kDa (AcTrxR-L) usually found in mammals (Leitsch et al., 2021). Their work focused on the recombinant expression and biochemical characterization of TrxR-S. However, in their study, Leitsch et al. (2021) attempted to express the enzyme AcTrxR-L using various expression systems, but these systems interpreted the UGA codon as a stop codon, resulting in a truncated, non-functional enzyme. Consequently, they hypothesized that it was a selenoprotein; however, they could not confirm this since intricate synthesis machineries are responsible for the biosynthesis of selenoproteins, the recombinant expression of TrxR-L was not achieved and thus, its role not unraveled. This finding highlighted the need for an expression system where the UGA codon could be correctly interpreted as selenocysteine and confirm it.

Given the importance of cellular redox systems, their potential druggability in other parasites and the unknown role of TrxR-L, the aim of this study was to address if AcTrxR-L is essential to the amoeba and might represent a valid drug target. For that, the recombinant expression and biochemical characterization of AcTrxR-L as a selenoprotein was achieved following recently developed methods (Cheng et al., 2021). Its susceptibility to the gold-based compound auranofin, approved by the US FDA for the treatment of rheumatoid arthritis (Roder and Thomson, 2015), was evaluated. Two new compounds, thioredoxin reductase inhibitor −1 and −2 (TRi-1 and TRi-2), which are more specific against selenoprotein TrxR (Sabatier et al., 2021), were also studied. Moreover, the cytotoxicity of the respective compounds on human corneal epithelial cells was assessed.

## 2 Materials and methods

### 2.1 Expression and purification of recombinant AcTrxR-L and Trx

**Fig. 1:**
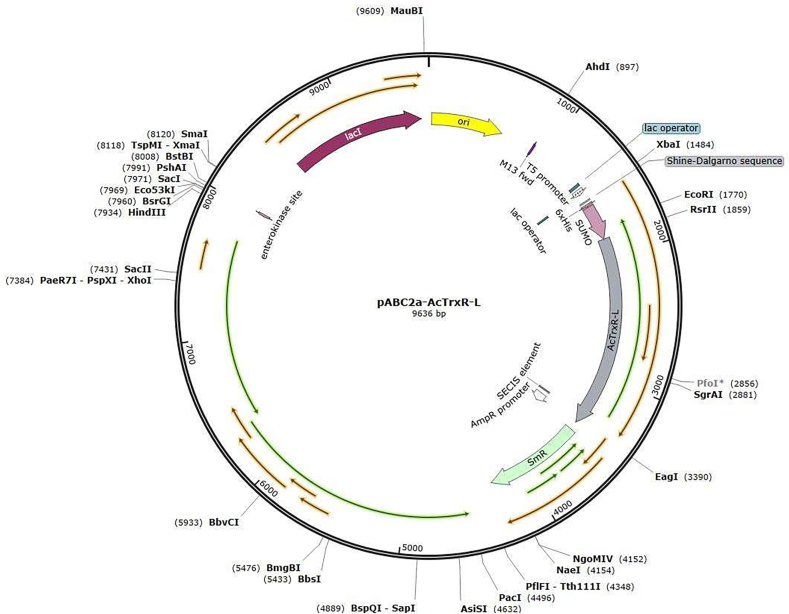
Scheme of the expression vector pABC2a containing His and SUMO motif and AcTrxR-L in the open reading frame and including a SECIS element.

**Fig. 2:**
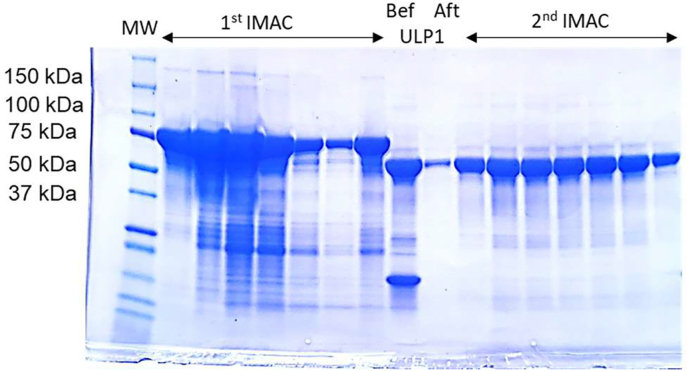
SDS-PAGE gel to assess the AcTrxR-L purification and purity. MW: molecular weight markers, as indicated. 1st IMAC: Elution fractions with the fusion target protein seen around 75 kDa before (Bef) and after (Aft) further purification upon digestion with ULP1: Target AcTrxR-L seen as band around ca. 55 kDa and the N-terminal His-tagged SUMO protein seen as a lower band. 2nd IMAC: Fractions containing the purified selenoprotein AcTrxR-L that were subsequently pooled and used for kinetic analyses. For further details, see the Methods description.

The AcTrxR-L gene (XP_004353633/ACA_1153040) was ordered from Eurofins and cloned into pABC2a expression vector, designing the protein fused with an N-terminal His- and SUMO motif (Fig. 1 and Table 1) using UAG together with a SECIS element for selenocysteine insertion (Cheng et al., 2021). The plasmid (pABC2a-AcTrxR-L) was subsequently transformed into *Escherichia coli* strain C321.ΔA for selenoprotein expression, followed by purification, tag removal and storage performed as previously described (Cheng et al., 2021; Cheng and Arnér, 2022). In short, elution of the target protein from a HisPrep FF 16/10 column equipped on an ÄKTA explorer FPLC system (Cytiva Life Sciences) was done with a mixture of 40% IMAC eluting buffer (approx. 200 mM imidazole final concentration) and 60% IMAC washing buffer. SUMO protease ULP1 was added to the fractions of the eluent with fusion protein and the mixture was carefully transferred into a pretreated dialysis tube which was sealed and dialyzed in 5 L dialyzing buffer overnight at 4 °C, allowing for ULP1 digestion. The dialyzed mixture was re-applied onto the HisPrep FF 16/10 column to separate cleaved non-tagged target selenoprotein from the N-terminal His-tagged fusion partner as well as from His-tagged ULP1. AcTrxR-L in the flowthrough fractions were collected and concentrated. The purity of the final selenoprotein AcTrxR-L was greater than 95% as assessed by SDS-PAGE (Fig. 2) and its concentration was 13.049 mg/ml calculated by measuring the absorbance at 463 nm on a spectrophotometer and using the extinction coefficient of FAD (AcTrxR-L is a homodimer with one FAD molecule in each subunit), which is 11,300 M^−1^cm^−1^ (Cheng and Arnér, 2022).

**Table 1:**
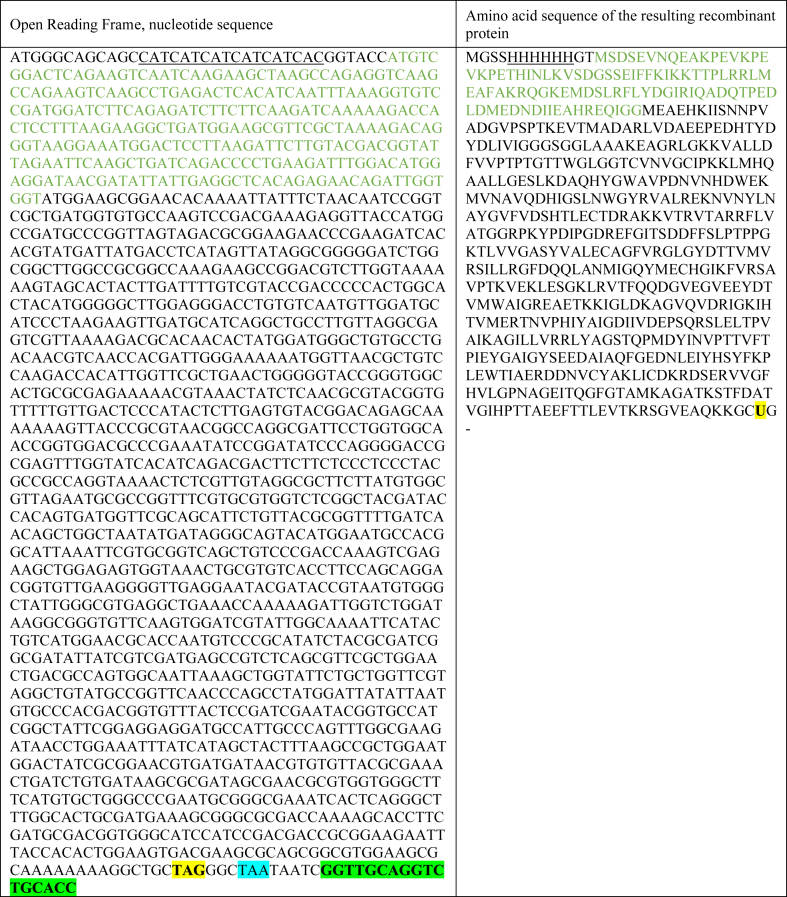
Sequences of the specific plasmid-encoded open reading frame (left column) and the resulting recombinant AcTrxR-L selenoprotein protein (right column). His-tag: underlined letters. SUMO sequence: green letters (cleaved off together with the N-terminal end during the purification procedure). Selenocysteine residue (U) and its codon (TAG): highlighted in yellow**.** Stop codon (TAA): highlighted in turquoise. C-terminal end of protein: hyphen (−). SECIS element (located 11 nucleotides downstream of the Sec-encoding TAG): highlighted in bright green.

**Fig. 3:**
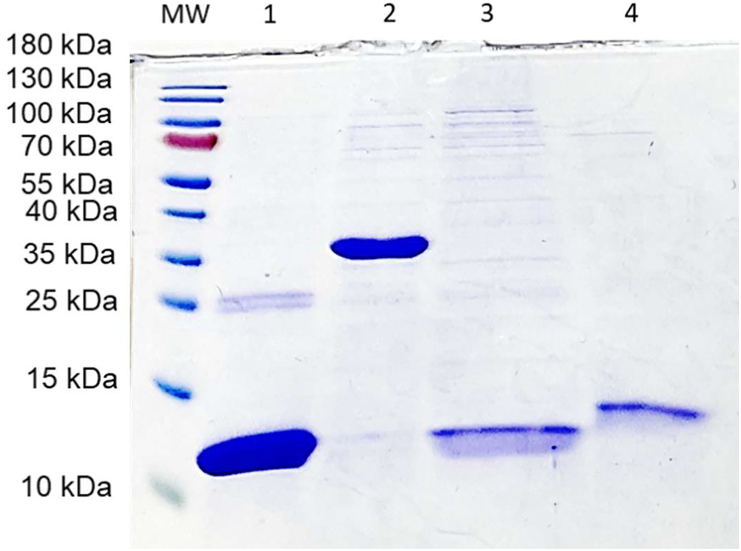
SDS-PAGE gel to assess the Trx purity. MW: molecular weight markers, as indicated. Lane 1: Purified recombinant AcTrx1. Lane 2: Purified recombinant AcTrx2. Lane 3: Purified recombinant NgTrx1. Lane 4: Purified recombinant NfTrx1.

To evaluate the enzymatic activity of AcTrxR-L and assess whether, like other selenoprotein TrxR enzymes, it is able to reduce a wide range of substrates, its activities were determined with *Acanthamoeba* thioredoxins (Trx), and the Trxs of another free-living amoeba, namely *Naegleria*, which are amoeboflagellates and are not phylogenetically related to the genus *Acanthamoeba* (Adl et al., 2005). The AcTrx1 (XP_004335509/ACA_1246790) and AcTrx2 (XP_004349558/ACA 1_322690) were cloned, expressed and purified as outlined previously (Leitsch et al., 2021). Thioredoxin-encoding genes for *N. gruberi* (NgTrx1-XP_002680876-) and *N. fowleri* (NfTrx1- XM_044703242-) were ordered from Eurofins and cloned into a pET-17b expression vector to be expressed with a C-terminal 6 x His tag in *E. coli* BL21-AI™ cells (Thermo Fisher). Expression was performed at 37 °C with vivid shaking for 3 h. After expression, *E. coli* cultures were spun down and lysed by grinding in a cold (−20 °C) mortar with a pestle. After removal of cell debris (20,000×*g*, 10 min), recombinant proteins were isolated in Ni-NTA spin columns (Qiagen) via the 6 x His tag, the purified protein was eluted in Buffer NPI-500 (Elution buffer for native conditions: 50 mM NaH_2_PO_4_, 300 mM NaCl, 500 mM imidazole, pH 8.0) and its concentration was measured by Bradford assay (Bio-Rad) (Bradford, 1976). All recombinant Trxs were isolated the day of the enzyme kinetic assays with their purities also assessed by SDS-PAGE gel (Fig. 3).

### 2.2 Enzyme kinetic assays with DTNB, GSSG, thioredoxin isoforms

Unless stated otherwise, all enzyme assays for AcTrxR-L were based upon well-established selenoprotein TrxR assays, as outlined earlier (Arnér, 2018). Kinetic parameters of AcTrxR-L were determined with different biochemical assays in triplicates in cuvettes as previously described (Fata et al., 2022). First, the specific activity of AcTrxR-L was calculated from its NADPH-dependent DTNB reduction. The reaction mixture contained 10 nM AcTrxR-L, 250 μM NADPH, and 2.5 mM DTNB in TE buffer (50 mM Tris and 2 mM EDTA, pH 7.5), and the reduction of DTNB was monitored by measuring the formation of the TNB^−^ anion at A412 nm on a spectrophotometer. Each DTNB is reduced into 2 molecules of TNB^−^ with extinction coefficient of 13,600 M^−1^cm^−1^ at 412 nm and the specific activity of the enzyme was calculated as Unit per mg (U/mg), with 1 U (μmol/min) defined as conversion of 1 μmol of DTNB substrate per minute.

Trx-coupled reduction of GSSG was measured in a reaction containing 50 nM AcTrxR-L, with or without 10 μM AcTrx1, in the presence of 1–5 mM GSSG and 250 μM NADPH, following NADPH consumption at A340 nm. The *G*lutat*h*i*o*ne as *s*ubstrate of *t*hioredoxin (GHOST) assay (Gromer et al., 2002) was used to test if the AcTrxR-L together with Trx variants would be able to indirectly reduce GSSG. The GHOST assay was carried out by measuring NADPH consumption using 50 nM AcTrxR-L, 250 μM NADPH, 2 mM GSSG and 0.1 μM–10 μM AcTrx1, AcTrx2, NgTrx1 or NfTrx1, and following A340. Experiments were performed in triplicates in cuvettes and enzyme velocity calculated based on a standard curve for NADPH (Gromer et al., 2002).

The activity of *Acanthamoeba* with different thioredoxin variants was assessed by measuring NADPH consumption linked to insulin reduction using 50 nM AcTrxR-L, 160 μM insulin, 250 μM NADPH and 1 μM–10 μM Trx variants (as indicated) following A340, the enzyme velocity calculated based on a standard curve for NADPH.

Apparent Michaelis-Menten parameters were in all cases calculated based on the nonlinear fit of the velocity vs substrate concentration curves to the Michaelis-Menten equation by using GraphPad Prism.

### 2.3 Inhibition of AcTrxR-L

The FDA-approved gold-based compound auranofin (Ridaura®), which inhibits the two mammalian selenoproteins TrxR and GPX1 (Sabatier et al., 2021; Cheff et al., 2023) and two new compounds that more specifically target the selenoprotein TrxR, known as thioredoxin reductase inhibitor −1 and −2 (TRi-1 and TRi-2) (Sabatier et al., 2021), were used to assess the inhibition of AcTrxR-L considering that other parasites selenoprotein TrxR enzymes, to this extent, can be inhibited by these compounds (Fata et al., 2022; Gencheva et al., 2022). The reaction consisted of incubating 50 nM AcTrxR-L with 0.5–10 μM of auranofin, TRi-1 or TRi- 2, or 1% v/v DMSO, 250 μM NADPH and 0.01 mg/ml BSA in TE buffer for 30 min (Fata et al., 2022). Then, the reaction was diluted 5-fold and 200 μl was desalted using Zeba™ Spin Desalting Columns and Plates, 40K MWCO (Thermo Scientific, 87767), whereupon 100 μl of the reactions, either desalted or not desalted, were transferred to each well of a 96 well plate. Subsequently, 250 μM NADPH and 1 mM DTNB were added to each well. The TNB^−^ formation at A412 nm was followed by a spectrophotometer and experiments were carried out in duplicates. The IC_50_ values were determined based on the nonlinear regression (curve fit), dose-response-inhibitor equation, log (inhibitor) vs. normalized response (variable slope) using GraphPad Prism.

### 2.4 *Acanthamoeba* growth conditions

*Acanthamoeba castellanii* strain Neff (ATCC® 30010™) genotype T4 was used for all experiments. The amoebae were grown in peptone yeast extract-glucose medium (PYG) in 75 cm^2^ tissue culture flasks at 25 °C with weekly medium changes as described previously (Köhsler et al., 2022).

### 2.5 *In vitro* amoebicide assays

The effects of auranofin, TRi-1 and TRi-2 were evaluated with the *Acanthamoeba* Neff strain. The amoebicidal activity was assessed by direct counting, using trypan blue (Martín-Pérez et al., 2021) and assayed in sterile 96-well microplates. Diverse drug concentrations (0–20 μM) were prepared via serial dilutions in DMSO, keeping total DMSO at 5% in the control well and in the drug solution wells ranging 5–3%. Amoebae from log-phase cultures (5 × 10^4^ trophozoites/well) were inoculated into the microplates and the trophozoites were resuspended in PYG medium. Drug assays contained 100 μL of trophozoites solution, 90 μL of PYG media and 10 μL of the drug solution per well. The growth control (positive control) included 100 μL of trophozoites solution, 90 μL of PYG medium and 10 μL of DMSO. Each drug concentration and control were investigated in triplicates and each experiment was repeated in two independent setups. The plates were incubated at 25 °C during 4, 24, 48 and 72 h. Samples were placed in a Bürker-Türk counting chamber. The IC_50_ was calculated using GraphPad Prism, as explained previously.

### 2.6 Cytotoxicity test on human corneal epithelial cells

Cytotoxicity of the compounds towards human corneal epithelial cells (HCECs) (Innoprot, ref. P10871) was determined (Loufouma Mbouaka et al., 2023) in 96-well microplates. The HCECs (1 × 10^4^ cells/well) were grown in 100 μL of corneal epithelial cell medium (CEpiCM) (Innoprot, ref. P60131) supplemented with 1% of corneal epithelial cell growth supplement (CEpiCGS), 2% of fetal bovine serum (FBS) and 1% antibiotic mix: 10,000 U penicillin, 10 mg streptomycin and 25 μg amphotericin B per milliliter. After overnight incubation, cytotoxicity was evaluated adding 20 μL of CellTiter 96® AQ_ueous_ One Solution Reagent (Promega, Madison, WI, USA). After 1 h of incubation at 37 °C, the absorbance at 490 nm was read using a spectrophotometer (Tecan, Spark 10M, Switzerland).

It was considered that viability values between 100 and 90% were non-toxic, 90–75% low toxicity, 75–60% moderate toxicity, and lower than 60% was considered high toxicity, as previously suggested (Lorenzo-Morales et al., 2010). The TD_50_ values were calculated using the same method as the IC_50_ values with GraphPad Prism. Moreover, the therapeutic index was determined as the ratio of TD_50_ to IC_50_ (Muller and Milton, 2012).

## 3 Results

### 3.1 Enzyme kinetic assays with DTNB, GSSG, thioredoxin isoforms

**Fig. 4:**
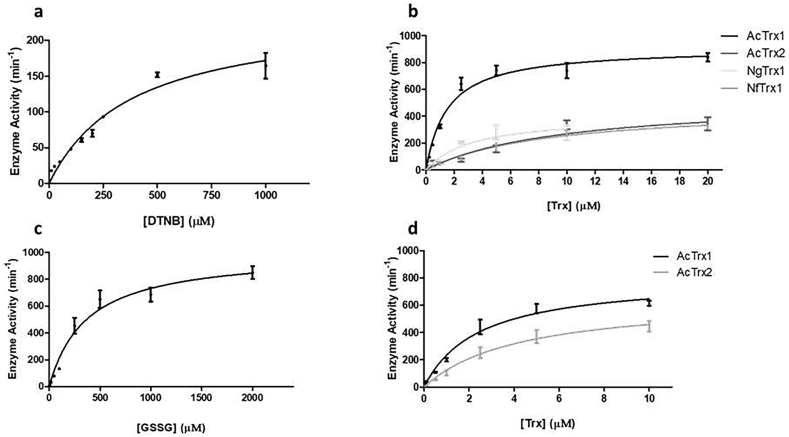
**Enzyme activities of AcTrxR-L. a.** Direct DTNB reduction assay. **b.** GHOST assay with different concentrations of the substrates AcTrx1, AcTrx2, NgTrx1 and NfTrx1 and constant concentration of GSSG. **c.** GHOST assay with different concentrations of GSSG and constant concentration of AcTrx1. **d**. Insulin reduction assay coupled with different concentrations of AcTrx1 and AcTrx2. For further details, see the Methods section.

The specific activity of the selenoprotein AcTrxR-L was 4.25 U/mg as calculated from the TNB^−^ formation in the regular DNTB assay (Arnér, 2018). The purified recombinant AcTrxR-L was found to exhibit substantial activity not only with DTNB (Fig. 4a) but also different parasite thioredoxins as coupled with GSSG reduction in the so called GHOST assay (Gromer et al., 2002) (Fig. 4b and c), or insulin (Fig. 4d). Table 2 summarizes the apparent kinetic parameters of the enzyme with these substrates.

**Table 2:** Kinetics of recombinant AcTrxR-L with different substrates.

Kinetic assay	Substrate	Active site of Trx substrate	Km (μM)	kcat (min^−1^)	kcat/Km (min^−1^ μM^−1^)	Vmax (μmol min^−1^ mg^−1^)
**GHOST assay**	**AcTrx1-coupled with GSSG**	-CGPC-	1.53 ± 0.21	18,234	11,9 × 10^3^	911.7 ± 34.95
	**AcTrx2-coupled with GSSG**	-CGPC-	9.69 ± 4.19	10,606	1,09 × 10^3^	530.3 ± 100.4
	**NgTrx1 coupled with GSSG**	-CGPC-	3.25 ± 1.40	8104	2,49 × 10^3^	405.2 ± 71.49
	**NfTrx1 coupled with GSSG**	-CSPC-	8.58 ± 1.69	9502	1,11 × 10^3^	475.1 ± 44.38
	**GSSG coupled with AcTrx1**		368.1 ± 70.11	20,040	0,054 × 10^3^	1002 ± 59.66
**Direct DTNB reduction assaay**	**DTNB**		384.1 ± 63.74	4762	0,012 × 10^3^	238.1 ± 19.01
**Insulin assay**	**AcTrx1 coupled with insulin**	-CGPC-	2.43 ± 0.46	16,072	6,61 × 10^3^	803.6 ± 56.05
	**AcTrx2 coupled with insulin**	-CGPC-	4.23 ± 1.40	13,062	3,09 × 10^3^	653.1 ± 96.29

### 3.2 Inhibition of AcTrxR-L

**Fig. 5:**
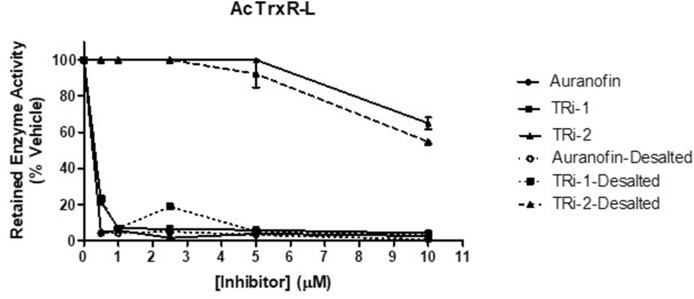
Relative enzyme activity of AcTrxR-L after 10 min of incubation with inhibitors. The resulting enzyme activity was determined in the direct DTNB reduction assay following A412. Error bars represent standard deviations of the mean. Solid lines represent the relative enzyme activity in the presence of the inhibitors and dashed lines after desalting.

In order to assess the potential for inhibition of AcTrxR-L, three compounds previously known to act as inhibitors of mammalian (Sabatier et al., 2021), *Schistosoma mansoni* (Kuntz et al., 2007) and additional (Fata et al., 2022) parasite selenoprotein thioredoxin reductase enzymes, namely auranofin, TRi-1 and TRi-2, were evaluated. Auranofin and TRi-1 were found to be very efficient inhibitors while TRi-2 showed less potency, as assessed with the direct DTNB reduction assay after 10 min incubation (Fig. 5). After removal of auranofin, TRi-1 and TRi-2 from solution by desalting, it was observed that AcTrxR-L was still inhibited to a similar extent, suggesting that all three compounds irreversibly inhibit the enzyme (dashed lines in Fig. 5). These observations were corroborated by the calculated IC_50_ values (Table 3). The IC_50_ values for auranofin and TRi-1 were identical even after the compounds were removed, confirming that these compounds irreversibly inhibit the enzyme. In contrast, the IC_50_ value for TRi-2 was higher than the concentrations studied in this assay.

**Table 3:** IC_50_ Values of AcTrxR-L using known selenoprotein thioredoxin reductase inhibitors. Not desalted values correspond to the IC_50_ values in presence of the inhibitors. Desalted values correspond to the IC_50_ values in absence of the inhibitors.

IC_50_ (μM)	Not desalted	Desalted
**Auranofin**	0.32 ± 0.39	0.32 ± 0.55
**TRi-1**	0.36 ± 0.07	0.37 ± 0.11
**TRi-2**	>10	>10

**Table 4:** IC_50_ values for the inhibitors tested against *Acanthamoeba* strain Neff trophozoites after different time points of treatment.

	IC_50_ (μM)			
	4h	24 h	48 h	72 h
**Auranofin**	209.80 ± 1.862	170.80 ± 4.623	21.09 ± 10.994	5.64 ± 1.346
**TRi-1**	71.04 ± 0.327	17.68 ± 1.645	7.65 ± 1.711	4.29 ± 0.862
**TRi-2**	226.1 ± 4.278	87.72 ± 7.441	4.18 ± 0.866	2.08 ± 0.489

**Table 5:** TD_50_ values for the inhibitors tested against human corneal epithelial cells after different time points of treatment.

	TD_50_ (μM)		
	24 h	48 h	72 h
**Auranofin**	2.79 ± 0.076	1.18 ± 0.103	0.97 ± 0.045
**TRi-1**	8.84 ± 0.044	8.40 ± 0.168	8.40 ± 0.003
**TRi-2**	11.17 ± 0.005	7.67 ± 0.363	8.08 ± 0.043

### 3.3 *In vitro* efficacy assays

**Fig. 6:**
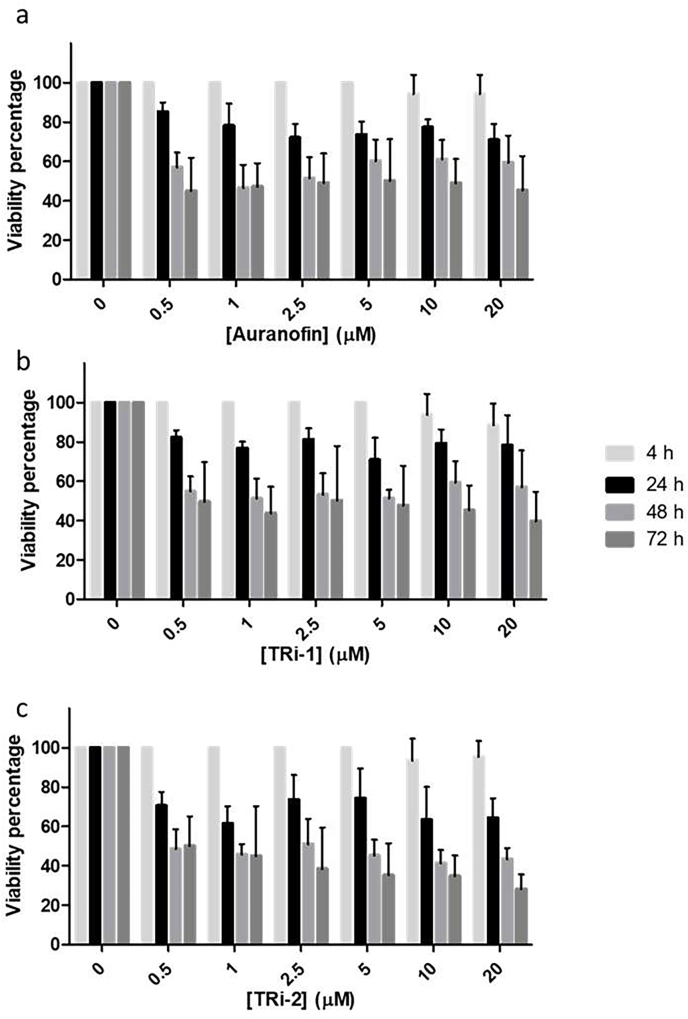
*Acanthamoeba* strain Neff viability percentage after treatment with the different compounds at different time points. **a.** Viability of trophozoites after treatment with auranofin. **b.** Viability of trophozoites after treatment with TRi-1. **c.** Viability of trophozoites after treatment with TRi-2.

The inhibitors of AcTrxR-L evaluated in this study were next evaluated for their efficacy against trophozoites in culture. To assess trophozoite viability, microscopic observations using the vital dye trypan blue were performed, as this method stains the dead trophozoites and confirms that the effect of the compounds are irreversible. The trophocidal activity of all compounds was found to be both, time and concentration dependent, with a decrease in viability percentage was observed with longer incubation times. However, for auranofin at a concentration of 1 μM, the viability percentages at 48 and 72 h were very similar, and the same pattern was observed for TRi-2 at a concentration of 0.5 μM (Fig. 6). The most effective compound was TRi-1, particularly at early time points, while TRi-2 showed the highest effectiveness after 48 and 72 h (Table 4).

### 3.4 Cytotoxicity test on human corneal epithelial cells

**Fig. 7:**
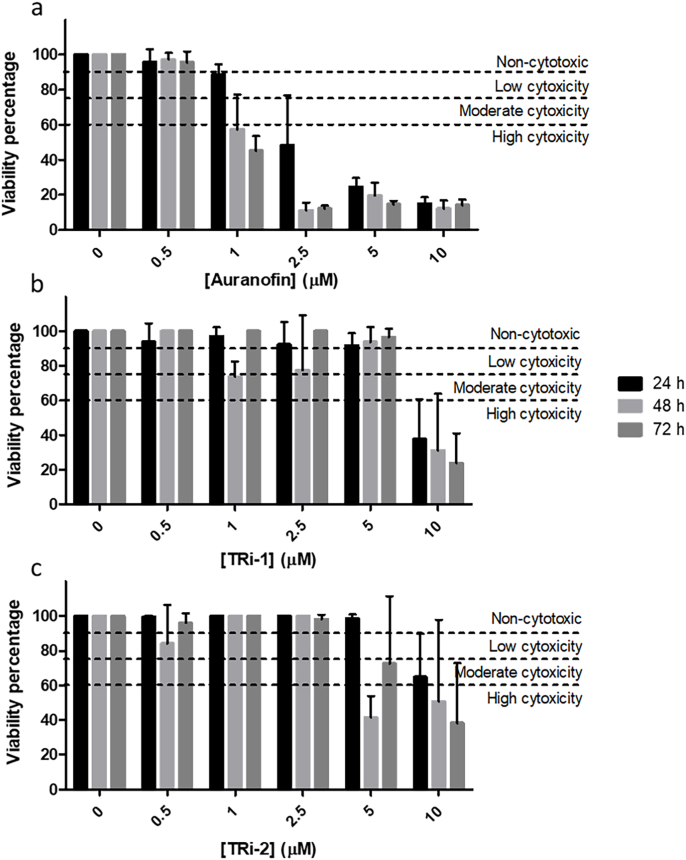
Viability percentages of human corneal epithelial cells (HCECs) after treatment with the different inhibitors at various time points. The indication of the degree of cytotoxicity is represented as previously suggested (Lorenzo-Morales et al., 2010). **a.** Viability of HCECs after treatment with auranofin. **b.** Viability of HCECs after treatment treated with TRi-1. **c.** Viability of HCECs after treatment treated with TRi-2.

The cytotoxicity profiles of the inhibitors were next tested against human corneal epithelial cells (HCEC), revealing that auranofin showed the highest cytotoxicity while all compounds were cytotoxic at higher (>5 μM) concentrations (Fig. 7). The TI was calculated (Table 6), revealing that TRi-2 has the highest TI (the larger the TI, the safer the drug). However, the cytotoxicity against *Acanthamoeba* strain Neff trophozoites was clearly more pronounced (compare Table 4, Table 5), suggesting a potential therapeutic window.

**Table 6:** Therapeutic index (TI) of the inhibitors tested after different time points of treatment.

	TI		
	24 h	48 h	72 h
**Auranofin**	0.02	0.056	0.17
**TRi-1**	0.5	1.10	1.96
**TRi-2**	0.13	1.83	3.88

## 4 Discussion

In this study, the recombinant expression of the large selenoprotein thioredoxin reductase (AcTrxR-L) of *Acanthamoeba* was achieved using the method developed by Cheng et al. (2021) for recombinant selenoprotein production using UAG as the Sec codon in a specific strain of *E. coli* (C321.ΔA), which lacks other UAG codons and the release factor RF1 that normally terminates translation at UAG, enabling its production in *E. coli* at a higher yield and purity. This production of AcTrxR-L enabled evaluation of its kinetic parameters and susceptibility to inhibitors as reported herein.

The selenoprotein AcTrxR-L, as discussed by Leitsch et al. (2021), is a part of the rather peculiar thioredoxin system in *Acanthamoeba*, with concurrent presence of GR and two TrxRs, one with a low molecular weight akin to that observed in bacteria (TrxR-S) and another being the high molecular weight selenoprotein AcTrxR-L resembling the orthologue found in vertebrates (TrxR-L). Recent studies (Köhsler et al., 2022; Leitsch et al., 2021) have shown that oxidative stressors enhanced the mRNA expression of GR but had no influence on protein levels. Moreover, GR showed high activity when it was measured in cell extracts. In the case of AcTrR-S, its activity could not be measured in the cell extract, but the expression levels in untreated cells were low, while in cells treated with oxidative stressors, the expression at mRNA and protein level was higher. However, AcTrxR-S showed strong activity with some substrates (AcTrx-1 XP_004335509/ACA_1246790). In these previous studies, the mRNA and protein expression levels of AcTrxR-L were low and did not change after exposure to oxidative stress. Therefore, the authors suggested that it is unlikely that AcTrxR-L is involved in antioxidative defense under normal growth conditions. Furthermore, studying AcTrxR-L enzymatic activity with various substrates was not feasible at that time, as it could not be produced (Leitsch et al., 2021). In the current study, the enzymatic activity was successfully analyzed and it was shown, that while AcTrxR-L can reduce AcTrx-1 and AcTrx-2 (XP_004349558/ACA 1_322690), its affinity for these substrates is not as high as that of AcTrxR-S. In addition, it was observed that it is also able to reduce Trx from other free-living amoebas, including *Naegleria fowleri* and *N. gruberi*, although they are not phylogenetically close; the affinity shown for these Trxs (NfTrx1 - XM_044703242 and NgTrx1 - XP_002680876) was slightly lower than that for AcTrx1 and AcTrx2. Interestingly, AcTrxR-L was here shown to be able to reduce GSSG when coupled to AcTrx1, similarly to the case with the TrxRs of the filarial nematodes *Brugia malayi* and *Onchocerca volvulus* (Fata et al., 2022), as well as human TrxR1 (Gromer et al., 2002). This indicates that AcTrxR-L together with AcTrx1 can substitute for TrxR-S or GR functions. Given its capacity to reduce Trx from other species, it is conceivable, as other authors have hypothesized, that it plays a role in pathogenicity and is involved in the oxidative response produced by host immune system cells (Leitsch et al., 2021). Nevertheless, additional research is required to fully understand the role of AcTrxR-L *in vivo*.

In recent studies, auranofin was also found to effectively inhibit the enzymatic activity of AcTrxR-S, with an IC_50_ of 216 nM (Leitsch et al., 2021). When *A. castellanii* trophozoites were treated with auranofin, both of the AcTrxR-S and AcTrxR-L mRNA expression levels significantly decreased, while GR mRNA levels increased (Köhsler et al., 2022; Leitsch et al., 2021). In addition, auranofin exhibited a strong amoebicidal effect against different strains of *Acanthamoeba*, with an IC_50_ ranging from 2.9 to 3.48 μM after 72 h of treatment (Loufouma Mbouaka et al., 2021). In the current study, an efficient auranofin-mediated inhibition of AcTrxR-L was demonstrated, and using desalting also shown to be irreversible. The efficacy of auranofin against trophozoites of the *Acanthamoeba* Neff strain after 72 h of treatment was lower (IC_50_ 5.64 μM) than that reported by Loufouma Mbouaka et al. (2021), but similar to the one observed by Feng et al. (2020) – 5.79 μM. Together, these observations show that auranofin has pleiotropic effects towards the amoeba redox systems, resulting in amoebicidal efficacy.

Here, the potential inhibitory effects of the novel compounds TRi-1 and TRi-2, which inhibit more specifically than auranofin selenoprotein TrxR enzymes, were also investigated. While TRi-2 did not completely suppress the enzyme activity of AcTrxR-L at any of the tested concentrations, TRi-1 demonstrated an inhibitory capacity equivalent to that of auranofin. Also the inhibitions by TRi-1 and TRi-2 were irreversible, as judged from the desalting experiments. These compounds have also been shown to inhibit the selenoprotein orthologues of *Brugia malayi* and *Onchocerca volvulus* (Fata et al., 2022). TRi-1 successfully inhibited those enzymes at the same level as auranofin, and TRi-2 was less effective (Fata et al., 2022). Surprisingly, when *A. castellanii* Neff trophozoites were treated with these compounds, TRi-2 (IC_50_ 2.08 μM) was more effective than the other two inhibitors and TRi-1 (IC_50_ 4.29 μM) reduced trophozoite viability to the same extent as auranofin. Hence, the ability of TRi-2 to kill trophozoites does not correspond to its capacity to inhibit AcTrxR-L, so it is plausible that this compound may also affect other critical molecules in the *Acanthamoeba* metabolism. Naturally, both uptake and intracellular metabolism can also differ between the compounds as other reasons for divergent amoebicidal profiles. Additionally, in this study, the effectiveness of the compounds was tested only in the Neff strain (since it is well-characterized and commonly used in research), but the effects of the compounds may be differ between clinical isolates. Further assays with several clinical isolates will be carried out in the future to understand the effect of the compounds, as well as, the characterization of the enzyme AcTrxR-L in the different clinical isolates.

The cytotoxicity of auranofin, TRi-1 and TRi-2 was also studied in human corneal epithelial cells (HCEC) showing that all of them are cytotoxic to some extent; however, in the cases of TRi-1 and TRi-2 (IC_50_ 8.40 and 8.08 μM, respectively), the toxicity towards *Acanthamoeba* trophozoites was considerably higher, indicating that less concentration would be needed to kill trophozoites than to induce damage to HCEC. Moreover, chlorhexidine, the drug of choice in AK treatment, has an IC_50_ of 1.58 μM against HCEC, thus exerting even higher cytotoxicity (Martín-Pérez et al., 2019). Therefore, these compounds are valid drug candidates and should not be discarded as future amoebicidal agents, either in the treatment of AK or as a disinfectant in contact lens solutions, given their favorable therapeutic index.

Research on amoeba antioxidant enzymes as potential drug targets has been intensified in the past few years (Andrade and Reed, 2015; Leitsch et al., 2018). Most organisms have two parallel systems that regulate redox balance: one based on glutathione (GSH) and the other on isoforms of thioredoxin (Trx). These systems are essential for maintaining redox homeostasis and are involved in vital physiological processes (Arnér, 2022). In several parasites, these enzymes have been successfully inhibited and proposed as drug targets. For example, the inhibition of the trypanothione reductase in trypanosomatids has a parasiticidal effect (Krauth-Siegel et al., 2005). An antiparasitic effect was also observed after silencing the thioredoxin glutathione reductase (TGR) expression in *Schistosoma mansoni*, and the treatment with auranofin in infected mice reduced the worm burden by 60% (Kuntz et al., 2007). In the present study, we have shown that the inhibition with auranofin, TRi-1 and TRi-2 led to the death of *Acanthamoeba* trophozoites. However, the unique system of *Acanthamoeba* with three enzymes involved in the redox regulation (AcGR, AcTrxR-S and AcTrxR-L) increases the difficulty of their use as therapeutic targets since the amoeba has several backup systems. Further studies are needed to determine how the three enzymes work in combination and to develop a strategy to inhibit them effectively to kill *Acanthamoeba* trophozoites.

## 5 Conclusions

In summary, this study provides a detailed potential analysis of targeting the large thioredoxin reductase (AcTrxR-L) as a novel therapeutic strategy for *Acanthamoeba* infections. Moreover, the inhibitory effects of auranofin, TRi-1, and TRi-2 on AcTrxR-L activity were assessed, with auranofin exhibiting potent inhibition followed by TRi-1, indicating that the inhibition of AcTrxR-L affects the viability of *Acanthamoeba* trophozoites, suggesting their potential as future amoebicidal agents. Further research into its structure, function, and inhibition mechanisms is needed to fully exploit its therapeutic potential.

## CRediT authorship contribution statement

**Alvie Loufouma-Mbouaka:** Writing – review & editing, Investigation. **Attila Andor:** Writing – review & editing, Methodology, Investigation, Data curation. **David Leitsch:** Writing – review & editing, Methodology, Conceptualization. **Jorge Pérez-Serrano:** Writing – review & editing, Methodology. **Elias S.J. Arnér:** Writing – review & editing, Resources, Methodology, Funding acquisition, Conceptualization. **Julia Walochnik:** Writing – review & editing, Resources, Conceptualization. **Tania Martín-Pérez:** Writing – review & editing, Writing – original draft, Visualization, Validation, Supervision, Resources, Project administration, Methodology, Investigation, Funding acquisition, Formal analysis, Data curation, Conceptualization.

## Declaration of competing interest

E.S.J.A. is co-inventor and co-owner of patents on the TRi-1 and TRi- 2 compounds that have been licensed to a company developing them towards clinical applications.
